# Health care providers’ awareness on medical management of children with autism spectrum disorder: cross-sectional study in Russia

**DOI:** 10.1186/s12909-021-03095-8

**Published:** 2022-01-10

**Authors:** Y. O. Mukhamedshina, R. A. Fayzullina, I. A. Nigmatullina, C. S. Rutland, V. V. Vasina

**Affiliations:** 1 Clinical Research Center for Precision and Regenerative Medicine, Kazan Federal University, Kremlevskaya St 18, Kazan, 420008 Tatarstan Russia; 2 Department of Histology, Cytology and Embryology, Kazan State Medical University, Kazan, Russia; 3 Department of Propaedeutics of Pediatric Diseases and Faculty Pediatrics, Kazan State Medical University, Kazan, Russia; 4 Department of Psychology and Pedagogy of Special Education, Kazan (Volga region) Federal University, Kazan, Russia; 5 School of Veterinary Medicine and Science, University of Nottingham, Nottingham, UK

**Keywords:** Health care providers’ awareness, Autism spectrum disorder, Online survey

## Abstract

**Background:**

Autism spectrum disorder (ASD) is a complex developmental range of conditions that involves difficulties with social interaction and restricted/repetitive behaviors. Unfortunately, health care providers often experience difficulties in diagnosis and management of individuals with ASD, and may have no knowledge about possible ways to overcome barriers in ASD patient interactions in healthcare settings. At the same time, the provision of appropriate medical services can have positive effects on habilitative progress, functional outcome, life expectancy and quality of life for individuals with ASD.

**Methods:**

This online survey research study evaluated the awareness and experience of students/residents (*n* = 247) and physicians (*n* = 100) in the medical management of children with ASD. It also gathered the views and experiences of caregivers to children with ASD (*n* = 158), all based in Russia.

**Results:**

We have established that the Russian medical community has limited ASD knowledge among providers, and have suggested possible reasons for this. Based on results from online surveys completed by students/residents, non-psychiatric physicians, and caregivers of children diagnosed with ASD, the main problems pertaining to medical management of individuals with ASD were identified. Possible problem solving solutions within medical practice were proposed.

**Conclusions:**

The results from this study should be considered when implementing measures to improve healthcare practices, and when developing models for effective medical management, due to start not only in Russia but also in a number of other countries.

**Supplementary Information:**

The online version contains supplementary material available at 10.1186/s12909-021-03095-8.

## Background

Autism spectrum disorder (ASD) is the fastest growing developmental disorder [1]. Early intense and specific intervention for ASD leads to improved cognitive and adaptive functioning [2, 3], therefore early identification of a potential risk of ASD in young children is particularly important. Pediatricians, as well as other health care providers, working with young children and families must be prepared to use specific tools for developmental monitoring and to screen for ASD in their practice. Initially, in 2006 the American Academy of Pediatrics recommended screening specifically for ASD during well-child preventive care visits at 18 and 24 months old [4]. In contrast, in Russia a screening procedure for risk of ASD at the age of at least 24 months was only approved by a Ministry of Health order as recently as 2019 [5].

In addition to the above, one should take into consideration that ASD is a whole-body condition that affects not only the brain but also the functions of many organs and systems [6]. More than 80% of individuals with ASD are noted to have one or more co-morbid medical and neurological disorders [7]. The mortality rate of people with ASD is up to 10-fold higher than the general population [8, 9]. This higher mortality usually results from comorbidities such as epilepsy, gastrointestinal and respiratory disorders [10–13]. In addition, childhood functional impairments, and impairments in social reciprocity, were significant predictors of mortality [14]. Therefore, timely diagnosis, follow-up, and treatment of comorbidities by specialists capable of working with this group of patients can not only improve the quality of life of children with ASD, but also help save their lives.

Previously, health care providers were shown to have a limited level of knowledge and awareness concerning ASD screening and the monitoring/treatment of co-morbid conditions [15–17]. Medical students and non-neuropsychiatric disciplines residents have generally exhibited limited awareness regarding the topic of childhood ASD, as indicated by other studies [18–20]. The consequences of the above findings can lead not only to delays in early interventions, which endanger productive results, but they also contribute to the deterioration of the somatic state of the health of children with ASD [21]. There have not been previous studies into the awareness of medical management of children with ASD in health care providers in Russia. However, this data is of importance in order to identify where knowledge deficits lie and to provide useful suggestions, in the light of the study outcomes, for national and local management and training programmes and guidance.

## Methods

### Participants and procedure

All of the participants were informed verbally, or in writing, about the purpose and confidentially of the survey. The methods described herein were approved by the Kazan Federal University Local Ethics Committee and performed in accordance with relevant guidelines and regulations.

The medical students and residents group comprised of 247 people with an average age of 23.4 ± 2.2 years. Inclusion criteria were as follows: (1) participants were studying in their 5th or 6th year within a medical faculty or their 1st or 2nd year of residency, (2) participants were able to read, write, and understand Russian, (3) residents had to practice in non-neuropsychiatric disciplines, and (4) all participants had to be willing and able to participate in the study. The students and residents were invited to take part in an online survey by the dean’s office of their faculties between May and June, 2020.

A total of 100 Russian non-psychiatric physicians participated in this study. All of the physicians worked directly with children within their specialization areas, including pediatricians (50%), neurologists (34%), and medical specialists which consisted of ENT-specialists, surgeons, ophthalmologists and allergists (16%). The online survey was conducted between May and July, 2020, following invitations from management within the medical faculties and from professional associations. The professionals were relatively experienced in their professional roles: 20% had less than 5 years’ experience, 21% had 5–10 years’ experience, 14% had 10–15 years’ experience, 11 and 34% had 15–20 years’ and over 20 years’ experience, respectively.

Both surveys (designed for either students/residents or physicians) contained two sections and took approximately 5 min to complete (Supplementary materials S1). Part 1 comprised of 11–12 items on the participants’ backgrounds, including basic demographics (age, location and number of years in current practice, specialty and a type of care provided). Part 1 also contained questions relating to signs of ASD, its diagnostics and comorbidities, as well as information pertaining to completion of ASD patient management educational programs and the adequacy of knowledge gained through these courses. Part 2 was intended only for students/residents or physicians who had direct practical experience with patients with ASD (in Russia students start their medical practice in the 3rd year of their studies). Part 2 also summarized possible difficulties encountered in relation to diagnosis and follow-up of ASD comorbidities and their personal assessment of how to overcome them.

A total of 158 caregivers to children diagnosed with ASD were invited to participate in an online survey between April and June 2020. Attending physicians, or non-profit organizations supporting individuals with ASD and their families, highlighted the study to the participants. A questionnaire containing issues relating to a diagnostics procedure and the presence of comorbidities in children with ASD was used in the study (Supplementary materials S2). In addition, the query items included questions regarding satisfaction with the quality of medical care for children with ASD and related conditions, and the difficulties caregivers needed to overcome in obtaining service provision.

### Statistical analysis

Data obtained from the survey were imported into R 3.6.3 software (R Foundation for Statistical Computing, Vienna, Austria). Descriptive data are presented in absolute numbers and percentages. Qualitative analysis was carried out in relation to open-ended questions in the surveys. Participant responses were categorized and analyzed according to traditional methods of content analysis [22, 23]. Correlation analysis of the association between two ordered factors was performed using kendall coefficient correlation τb.

## Results

**Fig. 1:**
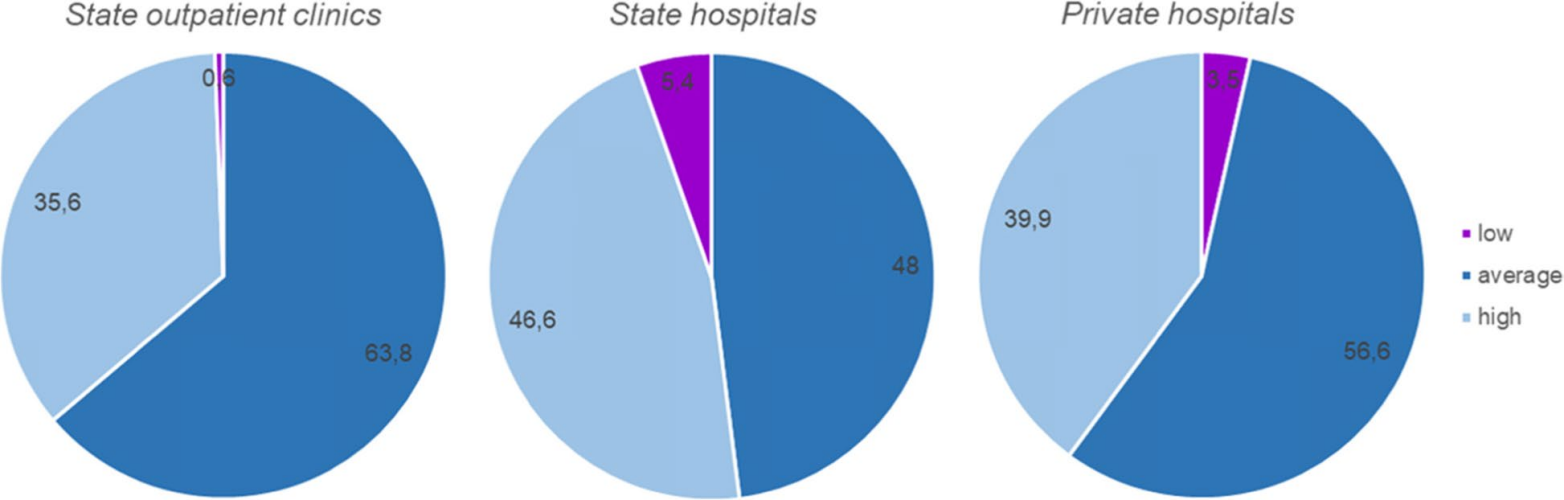
Quality evaluation of medical care provision in different medical facilities as reported by caregivers of children diagnosed with ASD (in %). Purple, dark blue and light blue indicated low, average and high satisfaction, respectively, of caregivers about the quality of medical care provided to their child with ASD

Our survey revealed that 62% of physicians and 17.8% of students and residents had experienced interactions with patients diagnosed with ASD during their medical practice. At the same time 80.6% of physicians and 63.6% of students and residents, from the above population of health care providers with previous experience with ASD, had difficulties in the diagnosis or follow-up of concomitant disorders for these patients. These factors also resulted in dissatisfaction with the quality of medical care for children with ASD, as reported by their caregivers (Fig. 1). Therefore, further consideration of the key difficulties in medical service provision and possible ways to overcome them seemed to be important and relevant.

### Limited provider knowledge

In Russia, based on our data, 63.5% of students and residents indicated that they did not study the signs and symptoms of ASD, and the special features of managing these patients, within their university/residency educational programs. It should be noted that medical faculty curriculums do contain ASD teaching; however, it is a 1.5-h lecture course without any seminars. Therefore, it was not surprising that the majority (74.2%) of non-psychiatric physicians in Russia who conduct clinical follow-ups of patients with ASD, considered that their knowledge relating to diagnostic criteria, especially early diagnosis, and on concomitant disorders and correct effective interactions with a child during an examination, to be inadequate.

Behavioral markers of ASD were considered by less than 50% of students/residents and physicians to be the leading clinical signs of ASD (Table 1). In total 66.6% of pediatricians indicated that deficits in social communication and interaction were one of the clinical signs of ASD, this reduced to 32.8% for other physicians. At the same time 30% of the respondents added non-specific clinical signs such as mental retardation and the lack of emotional empathy. Despite the established definitive criteria, 56.4% of pediatricians and ~ 35% of other respondents considered delayed speech or the lack of speech to be a leading diagnostic criterion of ASD. Around 10% of students/residents and ~ 20% of physicians considered limited eye contact and aggression to be clinical signs of ASD without knowledge about another specific clinical signs. In addition, our data showed that an increase in the number of years practicing did not have a positive correlation with awareness of the clinical signs of ASD. The above data were consistent with the results from responding caregivers who indicated that the predominant role in the detection of clinical signs of ASD is played by the parents themselves, and to a lesser extent by neurologists, but, unfortunately, not by pediatricians.

**Table 1:** Most cited clinical signs of ASD indicated by participants

***Clinical signs***		***Students/residents***	***Pediatricians***	***Other physicians***
DSM-5^a^ ASD Diagnostic Criteria	Persistent deficits in social communication and social interaction	49.6%	66.6%	32.8%
	Restricted, repetitive patterns of behavior, interests, or activities	42.5%	23.1%	36%
Delayed speech or absence of speech		35.2%	56.4%	34.4%
Limited eye contact		9.7%	20.5%	21.3%
Aggression/self-injury		10.5%	23.1%	13.1%
Mental retardation		17%	7.7%	5%
Lack of emotional empathy		6.5%	5.1%	6.5%
Do not know clinical signs of ASD (no answer/not a single correct answer)		17.4%	12.8%	24.6%

^a^*DSM-5* Diagnostic and Statistical Manual of Mental Disorders, 5th ed.; American Psychiatric Association, 2013

The Autism Diagnostic Observation Schedule (ADOS) and the Autism Diagnostic Interview-Revised (ADI-R) assessments are considered to be the gold-standard for diagnosing ASD. Although non-psychiatric physicians are not required to be proficient in ADOS (this is the prerogative of psychiatrists), they should still be aware of standard ASD diagnostic methods. Our data showed that only 16% of physicians and 18.6% of students/residents who participated in our survey knew about instruments such as ADOS for diagnosing and assessing ASD. Less than 7% of health care providers were aware of the possibilities of using ADI-R.

**Fig. 2:**
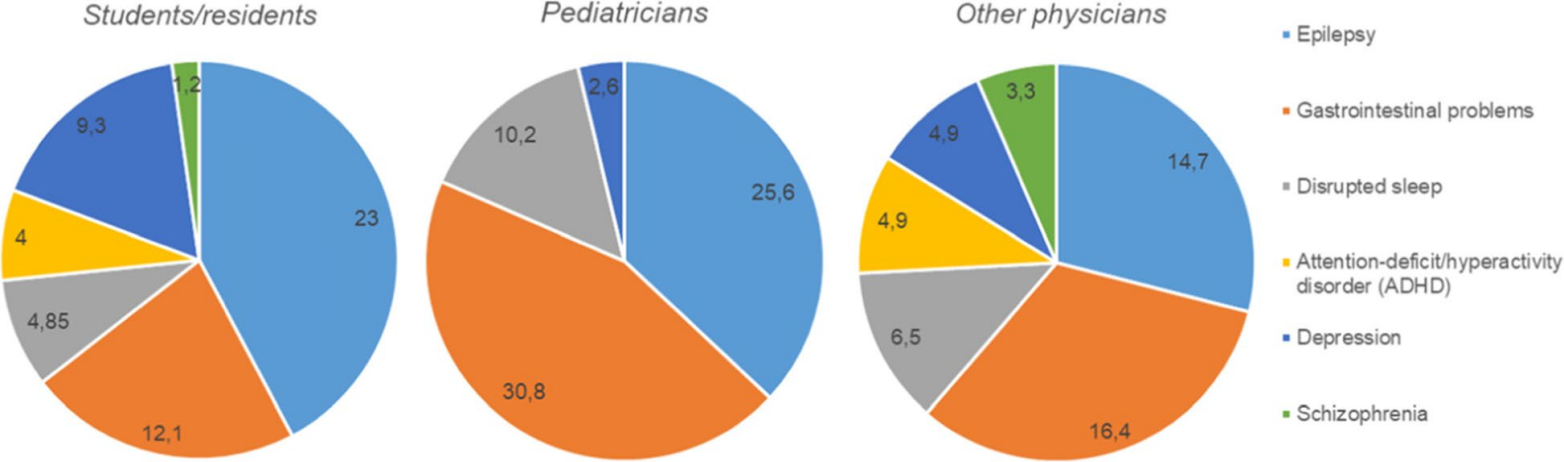
Most cited medical conditions associated with ASD indicated by participants (in %)

A total of 44% of physicians and 56.6% of students/residents who participated in our survey were unable to name any common concomitant disorders in individuals with ASD. Results from the health care provider survey investigating the most common comorbidities in patients with ASD are given in Fig. 2. It is worth noting, as in the case of awareness of clinical signs, an increase in the number of years practiced did not have a positive correlation with knowledge about ASD concomitant disorders.

**Fig. 3:**
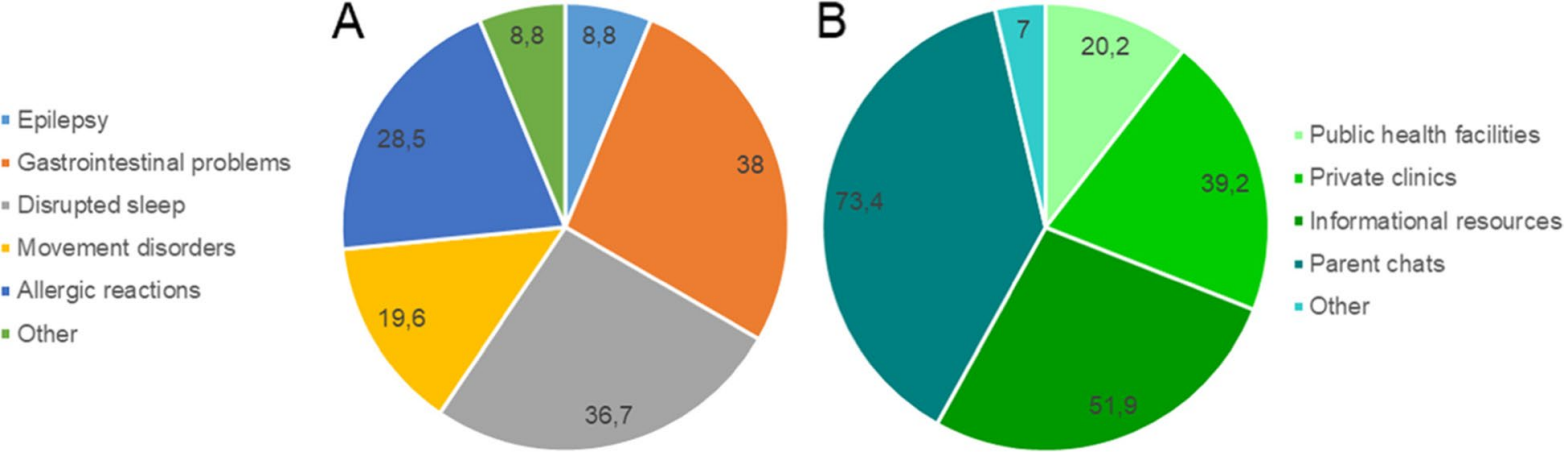
(**A**) The most cited concomitant disorders in children with ASD, and (**B**) the sources by which information relating to correction/therapy methods of concomitant disorders were obtained, as indicated by responding caregivers. Data are presented as a percentage

Given the information available from health care providers, it is not surprising that 46.2% of caregivers mentioned the lack of monitoring and correction/therapy of concomitant disorders by medical specialists for their children with ASD. A total of 37.3% of responding caregivers indicated that their children with ASD had one clinically proven concomitant disorder, and 26.6 and 15.2% had two or three or more concomitant disorders (Fig. 3А). Of particular interest were results relating to the sources of information obtained about the correction/therapy methods of concomitant disorders by caregivers of children diagnosed with ASD (Fig. 3B).

### Communication barriers

Our findings revealed that ~ 75% of students/residents and physicians related difficulties in diagnosis and follow-up of comorbidities to behavioral disorders only. At least 70% of the respondents mentioned the combination of the above problem with the lack of, or failed, language abilities and an inability to describe pain/discomfort experienced and their localization. At the same time only 2% of health care providers considered that additional information on effective methods of interaction with an ASD patient during an examination was required. It is worth noting that none of the medical participants trained in the diagnostics or medical support of patients diagnosed with ASD were trained abroad, training was received in Russia only. Communication difficulties with ASD patients arose regardless of numbers of years in practice and type of practice settings of the surveyed health care providers. These findings are in line with the caregivers opinions, at least 70% of whom stated that physicians who visited children with behavioral disorders lacked examination skills.

### Interdisciplinary interaction

The studies conducted showed that the work of an interdisciplinary team (medical-psychological and pedagogical follow-up) facilitates better provision of care coordination and holistic service for individuals with ASD [24–26]. However, only 2% of respondent health care providers reported having close cooperation(s) with psychological and pedagogical specialists. In addition not more than 10% of physicians referred, or recommended, patients to these specialists when identifying children with ASD. An enhanced numbers of years in practice did not lead to increased likelihood of forming close cooperative healthcare with interdisciplinary teams. The exception to this was in the rare cases where neurologists had long-term medical support for patients diagnosed with ASD. This lack of interdisciplinary interaction was further evidenced by the answers given by caregivers to children with ASD, 41.1% of whom reported inadequate post-diagnostic support including education on evidence-based methods of ASD correction.

### Need for systemic changes

A number of studies have indicated that systemic changes are required in order to improve the healthcare environment [27–29]. Our survey demonstrated that 83.5% of caregivers to children diagnosed with ASD considered that services on special follow-up of a child with ASD were required. At least 50% of the respondents mentioned that the environment (play grounds/rooms where a child can be distracted or sensory load be reduced) should be adapted for a successful health examination.

## Discussion

This survey-design research study aimed to clarify the main issues surrounding medical management of individuals with ASD in Russia, including monitoring and follow-up of comorbidities, by taking the opinions of both health care providers and also caregivers of diagnosed children into consideration. Diagnosis and follow-up of concomitant diseases (comorbidities) in individuals with ASD do involve several problems [30, 31], which we identified in our results and which need further discussion.

Over the past decade a large number of studies have shown that health care providers have moderate awareness and limited knowledge among health care providers (residents and physicians) on care provision and medical management of patients with ASD [16, 18, 29, 32–35]. Interestingly, these results were obtained not only in developing countries (Nigeria, Turkey), but also in developed ones (United States, Canada) with long histories of researching ASD and extensive experience of managing patients in this category. Unfortunately, our results also indicated limited ASD knowledge among health care providers in Russia. Behavioral markers of ASD, including persistent difficulties with social communication and interaction, the presence of restricted and repetitive behaviors, interests and/or activities, are not always considered as leading clinical signs by the medical community. In our study this was especially the case among non-psychiatric physicians, with the exception of a few pediatricians.

As for the diagnostic criteria related to ASD, it was clear that healthcare providers did not always have access to competent resources. The fact that some physicians and scientific schools in Russia still subdivide psychotic forms of ASD and consider them and schizophrenia as the same entity, further aggravates this problem. Diagnostic criteria of ASD have been reformed since it was first described and have changed up until the present day, therefore it is not surprising that there is still no consensus on diagnostic criteria of the disorder and its very essence among the medical community. This factor is noted to be a great obstacle in providing consistent assessment and determination of management strategies [36, 37]. Early in 2020, parent and medical communities discussed new clinical guidelines on ASD, as developed by the Russian Association of Psychiatrists. The main challenge was that the clinical guidelines are unscientific and based on out dated knowledge about ASD, as were approaches to its management and rehabilitation. These issues were solved, due to active participation of parent and professional communities, and in July 2020 the Russian Federation Ministry of Health approved new clinical guidelines on the management of children with ASD. These guidelines aimed to improve the quality of care of children with ASD and will be mandatory starting from 2022. Despite this, medical facilities still need to teach their specialists the new guidelines and have had to conduct additional educational activities since their approval.

Medical conditions associated with ASD have long been sufficiently studied worldwide [30, 38–46]. They predominantly include, but are not limited to, the following physical and mental-health conditions: disrupted sleep, gastrointestinal problems, epilepsy, immune problems and attention-deficit/hyperactivity disorder. However, the problem of comorbidities, as well as management methods for ASD complex behaviors, in relation to being important for effective medical care [47], are quite poorly addressed in Russian professional medical sources [48–51]. In Russia, activities to make physicians aware of modern studies clarifying possible mechanisms, symptoms and consequences of comorbidities in ASD, as well as methods to overcome communication barriers, are primarily conducted by non-profit organizations supported by various grant-providing systems. Recently, as the issue is so important, there have been increasing numbers of professional programs aimed at advanced training for non-psychiatric physicians (mainly pediatricians and neurologists). However, these programs are not always budget-funded and may not provide the full-time education required, that, in our opinion, makes them less available and reduces the quality of the educational program in terms of physicians being able to adequately master the topic.

We consider the limited ASD knowledge provided among the Russian medical community, as confirmed in our study, to be due to the following problems: (1) the educational program intended for physicians does not provide sufficient knowledge of ASD in multidisciplinary contexts or in the practical skills required; (2) no consensus is available in relation to ASD diagnostic criteria and the very essence of the disorder in the medical community; (3) clinical guidelines on the management of patients with ASD based on the modern diagnostic principles and treatment approaches for this condition have been quite recently approved and it will take some time for physicians to be taught them. Finding solutions to these problems is vital in order to overcome the overall lack of information within the Russian medical community.

Impairment in communication and social interactions are defined as the main symptoms of ASD, and is also one of the problems encountered by physicians trying to diagnose concomitant disorders. In addition, individuals with ASD are often unable to formulate complaints and may become increasingly agitated during physical examinations [52–55]. In turn, physicians may feel uncomfortable and prefer to ascribe symptoms of concomitant diseases to “common” manifestations of ASD or even avoid working with patients in this category at all [56]. Therefore, patients with ASD indicate low satisfaction in relation to patient-provider communication and low healthcare self-efficacy as a result [57–59]. The above can occur as a result of insufficient skills relating to the examination of individuals with evident behavioral disorders, indeed this has been shown previously [34, 56].

It has already been noted that health care providers can be unaware of the need to adjust their communication styles to be effective with patients with ASD - or are unable/unwilling to do so [60]. Many countries have started to contemplate what should be done in order to improve patient-provider communication [61–64]. In 2002, the American Board of Pediatrics began certifying developmental-behavioral pediatricians, whose activities involve diagnosis of developmental disabilities, identification of comorbidities, and the care assistance of these children. Results from the study conducted by Hansen et al., (2016) showed that the clinical practices of developmental-behavioral pediatricians allowed them to complete diagnostic evaluations for ASD and that the multiple components of assessment obtained aligned with existing guidelines [65]. In Russia, and other countries, multidisciplinary interaction is often suboptimal and physicians are frequently not aware of possible alternative communication methods or have no skills relating to behavior and response during medical examination, preventive methods, or those of desensitization used in examination of individuals with ASD [66].

Communication is a two-sided process, therefore it should be noted that the introduction of training on compliant physical exam and common health care procedures into the program of behavior manipulation of individuals with ASD is of importance, especially when coupled with training medical personnel in effective cooperation skills with autistic patients [67–69]. One must assume that two-sided steps would be successful and can improve the present situation.

Combined knowledge and competence of interdisciplinary team specialists provides optimal conditions for the successful development of a child, his/her adaptation and social interactions in the community, as well as allowing them to overcome difficulties including those related to the heterogeneity of disorders [66, 70, 71]. Unfortunately, the results obtained from our research indicated poor development of interdisciplinary interactions among specialists in Russia.

At the same time, given the high prevalence of concomitant diseases, the follow-up of individuals with ASD by different medical specialists is required. Margaret Bauman, a pediatric neurologist, developed the first multidisciplinary clinical program for complex care of individuals with ASD and other developmental disorders, called LADDERS (Learning and Developmental Disabilities Evaluation and Rehabilitation Services). The developed approach, under the LADDERS project based the Autism Treatment Network medical program, is a classic model to create progressive comprehensive medical care for children with ASD in many countries. In Russia the development of multidisciplinary medical follow-up of children with ASD started in 2019 via active participation of the non-profit organization the Center of Autism Problems. In this regard, there are great expectations for many pediatric physicians to become more aware of concomitant diseases, modern principles of their diagnosis and treatment approaches, and for them to be trained in working with ASD individuals.

We have also proposed possible solutions for the problem identified in present medical practice (Table 2). It should be noted that unlike a number of European countries and the USA, the care for individuals with ASD in Russia is still in its early days. Much depends upon the availability of financial and methodological support for the management of such children in the region. Nevertheless, the initiative and motivation of parents and specialists, which are usually determined by a personal relation to ASD, have greatly promoted the creation of specialized care to these children over the past decade. These range from evidence-based correction methods of this disorder to the implementation of a model of inclusive school and the approval of clinical guidelines on medical care of children with ASD.

**Table 2:** The main problems of medical management of individuals with ASD (including monitoring and follow-up of comorbidities) and possible problem solving in medical practice

***No.***	***Problems***	***Problem solving***
1	Difficulties in early ASD identification by primary care pediatricians and limited provider ASD knowledge in general	(1–3) Changes into the medical education program: extending the topics of child and adolescent mental health, adequate studying of ASD in a multidisciplinary context, the development of diagnostic routing protocols, training in practical skills of communication with ASD patients and their examination
2	Lack of adequate skills in examination of patients with evident behavioral disorders	
3	Poor understanding of the need to adjust communication styles to be effective with patients	
4	Lack of the proper development of competence and skills in identification, assessment and follow-up of concomitant diseases	(4–5) Conducting multidisciplinary thematic conferences and studies. Development of methodical and clinical recommendations on identification and treatment of concomitant diseases in patients with ASD with participation of a multidisciplinary working group.
5	Poor multidisciplinary cooperation of specialists	
6	Lack of specialist follow-up services for patients with ASD for successful health examination	(6–7) Changes in health care system legal regulations
7	Low adjustment of health care environments for patients with ASD	

Improvement in medical care can have a positive effect on rehabilitation progress and quality of life for patients with ASD [72]. This issue is more and more relevant and is heavily associated with challenges to medical service provision, as indicated by both healthcare providers and caregivers for children with ASD. In our opinion, the required system changes can only be implemented if the legal regulations of the health care system are changed. The multidisciplinary teams of specialists and active members of parent communities should be engaged in discussing proposed changes.

## Conclusions

The provision of proper medical services can have a positive effect on habilitative progress, functional outcome, quality and life expectancy of individuals with ASD. In addition to primary examinations, diagnostics and diagnosis, health care providers can provide important long-term medical care, support and educate families by guiding them towards interventions which are effective for patients with ASD [72]. Needless to say, understanding and acknowledging the gaps in health care service provision with this population, both by medical and parent communities, is required for effective development of ASD medical management. Therefore, the results of this survey should be considered when making measures to improve health care practices, and during the development of a model of effective medical management which is due to start in Russia and in a number of other countries.

## Supplementary Information

**Additional file 1.**

**Additional file 2.**

## Data Availability

The datasets used and/or analysed during the current study are available from the corresponding author upon reasonable request.

## Abbreviations

ASD: Autism spectrum disorder
DSM: Diagnostic and statistical manual of mental disorders
LADDERS: Learning and developmental disabilities evaluation and rehabilitation services
