# Supplementary material for: Health care providers’ awareness on medical management of children with autism spectrum disorder: cross-sectional study in Russia
Source: BMC Med Educ. 2022 Jan 10;22:29. doi: 10.1186/s12909-021-03095-8 (PMC8751116; doi:10.1186/s12909-021-03095-8)
Supplement: Supplementary file 1 — Additional file 1. [file 12909_2021_3095_MOESM1_ESM.docx]

**Health care providers’ awareness on medical management of children with autism spectrum disorder: cross-sectional study in Russia**

Mukhamedshina Y.O.^1,2*^, Fayzullina R.A.^3^, Nigmatullina I.A.^4^, Rutland C.S.^5^, Vasina V.V.^4^

^1^ Clinical Research Center for Precision and Regenerative Medicine, Kazan Federal University, Kazan, Russia

^2^ Department of Histology, Cytology and Embryology, Kazan State Medical University, Kazan, Russia

^3^ Department of Propaedeutics of Pediatric Diseases and Faculty Pediatrics, Kazan State Medical University, Kazan, Russia

^4^ Department of Psychology and Pedagogy of Special Education, Kazan (Volga region) Federal University, Russia

^5^ School of Veterinary Medicine and Science, University of Nottingham, United Kingdom

** corresponding author*

Yana Mukhamedshina, MD, PhD

Kremlevskaya St 18, Kazan, 420008,

Tatarstan, Russia

phone: +7-927-430-7511

mailto: [yana.k-z-n@mail.ru](mailto:yana.k-z-n@mail.ru)

Supplement material S1

**Online survey of non-psychiatric physicians**

Dear Colleagues! We ask you to take part in this survey to obtain summary data on the awareness of physicians regarding autism spectrum disorder (ASD) and the main problems of diagnosis and management of comorbidities in these patients. The survey is anonymous and will not take you more than 5 minutes. The questionnaires you filled out will make a significant contribution to the development of a roadmap for improving medical support for children with ASD.

1. *Enter your age*

______

1. *Specify your region of residence*

______

1. *Indicate your length of service as a doctor*

- less than 5 years

- from 5 to 10 years

- from 10 to 15 years old

- from 15 to 20 years old

- over 20 years

1. *Indicate your specialization*

- pediatrics

- neurology

- psychiatry

- surgery

- dentistry

- other _______

1. *What type of medical care do you provide?*

- stationary healthcare

- outpatient

- private consultation appointment

1. *Do you have an understanding of Autism Spectrum Disorder (ASD)?*

- yes

- no

1. *Specify the clinical signs of ASD*

______

1. *Do you know what the "gold standard" for diagnosing autism is? (if yes, please write the correct answer)*

- no

- yes______

1. *List the 5 most common comorbidities in patients with ASD*

______

1. *Have you attended educational programs for medical support of patients with ASD? (if you did, write the approximate number of hours and type of educational programs: lectures, workshops, professional training, self-education)*

- no

- yes ________

1. *If yes, do you think that the acquired knowledge is enough for you to apply it in practice? (if not enough, please write why?)*

- yes

- no________

1. *Have you ever interacted with a patient diagnosed with ASD in your medical practice?*

- yes

- no

*The following questions are for non-psychiatric physicians with any experience with patients diagnosed with ASD. If you do not have such experience, we are grateful for your answers to the previous 12 questions.*

1. *What kind of interaction did you have with the patient with ASD?*

- consultation

- diagnostics

- treatment

- other _______

1. *In your medical practice, have you ever referred a child to a child psychiatrist with suspected ASD?*

- no

- yes

1. *Do you participate in interdisciplinary interaction in the supervision of a patient with ASD?*

- yes, I work closely with specialists in the psychological and pedagogical profile
- no, but I direct or advise them to contact specialists with a psychological and pedagogical profile
- no

1. *Have you had any difficulties in diagnosing or managing comorbidities in a patient with ASD?*

- no

- yes

1. *If yes, what were the problems?*

- behavioral disorders

- absence or lack of formation of spoken language

- the inability to describe the pain / discomfort experienced and their localization

- lack of typical symptoms of diseases

- other ________

1. *Have you managed to overcome these difficulties over time in the course of your professional activity?*

- no

- partially

- yes

**Online survey of medical students and residents**

Dear students and residents! We are asking you to take part in this survey to obtain summary data on the awareness of future physicians about autism spectrum disorder (ASD) and the main problems of diagnosis and management of comorbidities in these patients. The survey is anonymous and will not take you more than 5 minutes. The questionnaires you filled out will make a significant contribution to the development of a roadmap for improving medical support for children with ASD.

1. *Indicate your year of study at university or residency, respectively*

______

1. *Enter your age*

______

1. *Specify your region of residence*

______

1. *Indicate your specialization awarded / received during the study at the university*

- pediatrics

- medical care

- medical and preventive work

- dentistry

- other _______

1. *Do you have an understanding of Autism Spectrum Disorder (ASD)?*

- yes

- no

1. *Specify the clinical signs of ASD*

______

1. *Do you know what the "gold standard" for diagnosing autism is? (if yes, please write the correct answer)*

- no

- yes______

1. *List the 5 most common comorbidities in patients with ASD*

______

1. *Have you studied the signs of ASD and the peculiarities of the introduction of such patients within the framework of the educational program of the university/residency? (if you did, write the approximate number of hours and type of educational programs: lectures, workshops, professional training, self-education)*

- no

- yes, in residency ________

- yes, in university________

1. *If yes, do you think that the acquired knowledge is enough for you to apply it in practice? (if not enough, please write why?)*

- yes

- no________

1. *Have you ever interacted with a patient diagnosed with ASD in your student or medical practice?*

- yes

- no

*The following questions are for students/residents with any experience with patients diagnosed with ASD. If you do not have such experience, we are grateful for your answers to the previous 11 questions.*

1. *What kind of interaction did you have with the patient with ASD?*

- consultation

- diagnostics

- treatment

- other _______

1. *In your student or medical practice, have you ever referred a child to a child psychiatrist with suspected ASD?*

- no

- yes

1. *Have you had any difficulties in diagnosing or managing comorbidities in a patient with ASD?*

- no

- yes

1. *If yes, what were the problems?*

- behavioral disorders

- absence or lack of formation of spoken language

- the inability to describe the pain / discomfort experienced and their localization

- lack of typical symptoms of diseases

- other ________

1. *Have you managed to overcome these difficulties over time in the course of your professional activity?*

- no

- partially

- yes
