# Supplementary material for: Health care providers’ awareness on medical management of children with autism spectrum disorder: cross-sectional study in Russia
Source: BMC Med Educ. 2022 Jan 10;22:29. doi: 10.1186/s12909-021-03095-8 (PMC8751116; doi:10.1186/s12909-021-03095-8)
Supplement: Supplementary file 2 — Additional file 2. [file 12909_2021_3095_MOESM2_ESM.docx]

**Health care providers’ awareness on medical management of children with autism spectrum disorder: cross-sectional study in Russia**

Mukhamedshina Y.O.^1,2*^, Fayzullina R.A.^3^, Nigmatullina I.A.^4^, Rutland C.S.^5^, Vasina V.V.^4^

^1^ Clinical Research Center for Precision and Regenerative Medicine, Kazan Federal University, Kazan, Russia

^2^ Department of Histology, Cytology and Embryology, Kazan State Medical University, Kazan, Russia

^3^ Department of Propaedeutics of Pediatric Diseases and Faculty Pediatrics, Kazan State Medical University, Kazan, Russia

^4^ Department of Psychology and Pedagogy of Special Education, Kazan (Volga region) Federal University, Russia

^5^ School of Veterinary Medicine and Science, University of Nottingham, United Kingdom

** corresponding author*

Yana Mukhamedshina, MD, PhD

Kremlevskaya St 18, Kazan, 420008,

Tatarstan, Russia

phone: +7-927-430-7511

mailto: [yana.k-z-n@mail.ru](mailto:yana.k-z-n@mail.ru)

Supplement material S2

**Online survey of caregivers of children on the autism spectrum**

Dear caregivers! We ask you to take part in the survey to obtain summary data on satisfaction with the quality of medical management of children with autism spectrum disorder (ASD). Your opinion is a very important and provides a defining moment in improving medical services for individuals with ASD. This online survey is confidential.

1. *Specify your region of residence*

______

1. *Specify the diagnosis of your child*

- Early childhood autism

- Atypical autism

- Asperger's Syndrome

- Sensorimotor alalia*, autism spectrum disorder

- Other _______

1. *Who was the first to suspect the diagnosis?*

- Psychiatrist

- Neurologist

- Pediatrician

- Other _______

1. *At what age was diagnosed?*

______

1. *Has your child had a disability diagnosed?*

- Yes

- No

1. *If yes, at what age?*

______

1. *If the disability has not been established, what is the reason?*

- Not have been a commission of medical and social expertise

- Did not want one

- Other ________

1. *Does your child have any of the following comorbid disorders?*

- Sleep disturbance

- Epilepsy

- Disorders of the gastrointestinal tract (constipation, diarrhea, abdominal pain)

- Movement disorders

- Allergic reactions

- Other ________

1. *Is your child's comorbidities monitored and corrected/treated by health care providers?*

- Yes

- No

- Not all comorbidities

1. *Where do you get information about the methods of correction / therapy of concomitant diseases?*

- Public health facilities

- Private clinics

- Informational resources

- Parent chats

- Other ________

1. *How do you assess the quality of medical care provided to your child in public outpatient clinics?*

- High

- Average

- Low

- Other __________

1. *Has your child ever had to undergo examination or receive medical care in an in-patient department?*

- Yes

- No

1. *If yes, in which in-patient department?*

- Surgical

- Pediatric

- Psychiatric

- Other __________

1. *How do you assess the quality of medical care provided to your child in public in-patient departments?*

- High

- Average

- Low

- Other __________

1. *Is your child a user of services in private clinics?*

- Yes

- No

1. *If yes, what services do you use?*

- Consultations with medical specialist

- Laboratory research

- Functional diagnostics (EEG, MRI)

- Therapeutic procedures

- Other ____

1. *How do you assess the quality of medical care provided to your child in private clinics?*

- High

- Average

- Low

- Other __________

1. *How much money does a family spend annually on services that are not covered by compulsory health insurance or in private clinics?*

- Less than 10 thousand rubles

- From 10 to 30 thousand rubles

- From 30 to 50 thousand rubles

- From 50 to 100 thousand rubles

- More than 100 thousand rubles

1. *What, in your opinion, hinders the effective medical support of your child?*

- lack of proper training of medical specialists in the field of early detection of ASD

- lack of diagnostic routing

- lack of competence in the detection, supervision and treatment of comorbidities in children with ASD

- lack of skills in examining children with behavioral disorders

- inadequate post-diagnostic support

- Other _________

1. *What conditions, in your opinion, are lacking for your child to improve the quality of their medical support?*

- services for the special support of a child with ASD

- high-quality multidisciplinary support

- Other _________

1. *Have you and your child encountered unethical behavior on the part of healthcare providers? (In the "Other" column, we ask you to quote the statements of medical workers that were unethical from your point of view)*

- Yes

- No

- Other ________

Authors provide these links for reviewers only:

* Sensorimotor alalia is regarded as a specific Russian-language term but it has no analogs in neurology and neuropsychology.
